# Designing for Care

**DOI:** 10.1007/s11948-023-00434-4

**Published:** 2023-04-25

**Authors:** Giovanni Frigo, Christine Milchram, Rafaela Hillerbrand

**Affiliations:** Institute for Technology Assessment and Systems Analysis (ITAS), Karlsruhe Institute of Technology (KIT), Karlsruhe, Germany

**Keywords:** Care ethics, Energy ethics, Care, Design, Value change, Energy systems, Community battery

## Abstract

This article introduces *Designing for Care* (D4C), a distinctive approach to project management and technological design informed by Care Ethics. We propose to conceptualize “care” as both the foundational *value* of D4C and as its guiding mid-level *principle*. As a value, care provides moral grounding. As a principle, it equips D4C with moral guidance to enact a *caring process*. The latter is made of a set of concrete, and often recursive, *caring practices*. One of the key assumption of D4C is a relational ontology of individual and group identities, which fosters the actualization of caring practices as essentially relational and (often) reciprocal. Moreover, D4C adopts the “ecological turn” in CE and stresses the ecological situatedness and impact of concrete projects, envisioning an extension of caring from intra-species to inter-species relations. We argue that care and caring can influence directly some of the phases and practices within the management of (energy) projects and the design of sociotechnical (energy) artefacts and systems. When issues related to “value change” emerge as problematic (e.g., values trade-offs, conflicts), the mid-level guiding principle of care helps evaluate and prioritize different values at stake within specific projects. Although there may be several actors and stakeholders involved in project management and technological design, here we will focus on the professionals in charge of imagining, designing, and carrying out these processes (i.e., project managers, designers, engineers). We suggest that adopting D4C would improve their ability to capture and assess stakeholders’ values, critically reflect on and evaluate their own values, and judge which values prioritize. Although D4C may be adaptable to different fields and design contexts, we recommend its use especially within small and medium-scale (energy) projects. To show the benefits of adopting it, we envisage the application of D4C within the project management and the technological design of a community battery. The adoption of D4C can have multiple positive effects: transforming the mentality and practice of managing a project and designing technologies; enhancing caring relationships between managers, designers, and users as well as among users; achieving better communication, more inclusive participation, and more just decision-making. This is an initial attempt to articulate the structure and the procedural character of D4C. The application of D4C in a concrete project is needed to assess its actual impact, benefits, and limitations.

## Introduction

Care is an essential element of human life that individuals and groups experience directly. At times as caregivers, others as care receivers, we live thanks to caring practices, mutualistic relationships, and constant dependencies. Though caring relations are sometimes chosen and other times unchosen, they contribute to determine the kind of persons we have been, are, and will be. This paper introduces *Designing for Care* (D4C),^1^ a distinctive approach to project management and technological design^2^ informed by Care Ethics (CE). We propose to conceptualize “care” as a value while “caring” as a process. Then, we conceive “care” not only as *the foundational value* of D4C but also as its *guiding moral mid-level principle*.^3^ As a value, care provides moral grounding and, as a principle, it equips D4C with moral guidance to enact a *caring process*. This process is made of a set of concrete, and often recursive, *caring practices *which make care visible and concrete.

In this paper, we adopt the definition of care devised by Hamington as “performed acts that promote the well-being and flourishing of others and ourselves based on knowledge and responsiveness to the one cared for” (2019, p. 92). This definition is indeed well compatible with the responsive and action-oriented character of both project management and design thinking. By indicating care as “performed acts” it is also consistent with our use of “caring practices” in this paper as well as with Fisher and Tronto’s famous general definition of care, which implies a broad notion of “activities”.^4^ Finally, we posit that caring is not only a human affair. Accordingly, we embrace the recent proposal of an “ecological turn” in care thinking (de la Bellacasa, 2011, 2017). Care should extend beyond human-to-human relations to include non-human beings and entities so that caring practices should apply to both intra- and inter-species relationships.

In the context of rapid technological innovation, the emergence of disruptive technologies, and several interwoven environmental crises, bringing attention to “care thinking” serves a double purpose. On the one hand, despite contemporary life is increasingly taking place within the “Technosphere” (Haff, 2014a, b; Hofstetter, 1998), focusing on care reminds us that technoscientific advancements should remain means for enhancing the well-being of people and non-human beings, whose concrete existences should remain ends in themselves. On the other hand, expressly concentrating on care puts focus on the relational nature of personal and group identities, which affirms the necessity of enhancing caring practices such as attentiveness, responsibility, competence, and responsiveness (Tronto, 1993, 2013). Our goal is to explore how CE can influence and perhaps improve debates in ethics of technology generally, and energy ethics specifically (Frigo & Hillerbrand, 2022). For these reasons, we propose an approach to the management of (energy) projects and the design of (energy) technologies and systems informed by CE.

Our proposal relates to current debates in Value Sensitive Design (VSD). Authors in this field posit that if certain values are recognized at the early stages of the design process, they can be potentially embedded into concrete artefacts and systems (van Wynsberghe, 2013; Davis & Nathan, 2015; van den Hoven et al., 2015; Friedman et al., 2017). Here, we follow van de Poel and Royakkers in defining values as “lasting convictions or matters that people feel should be strived for in general and not just for themselves to be able to lead a good life or realize a just society”, (2011, p. 72). Recently, van de Poel (2018) highlighted the issue of “value change” as a problem that is often overlooked in VSD. Van de Poel (2018) discusses different ways in which values may change: (1) the emergence of new values; (2) changes in what values are relevant for the design of a certain technology; (3) changes in the priority or relative importance of values; (4) changes in how values are conceptualized; (5) changes in how values are specified, and translated into norms and design requirements (van de Poel, 2018). As a possible solution, van de Poel proposes three technical features of sociotechnical artefacts and systems (i.e., adaptability, flexibility, and robustness). He suggests that “these features can be designed into products or systems so that they can better adapt to changing values in the later phases of the life cycle of a product or system” (p. 3).

Acknowledging reasonable values pluralism (Johannsen, 2021), here we do not aim to suggest that care should be the fundamental or most important value in design. Moreover, we neither propose to conceive “care” as a value to be embedded in project management and in technological design in the sense of VSD nor as the addition of a technical feature to overcome value change. Instead, we envision care as a foundational value of D4C and as a mid-level guiding principle for a set of caring practices that can influence the processes of managing projects and designing technologies.

**Fig. 1:**
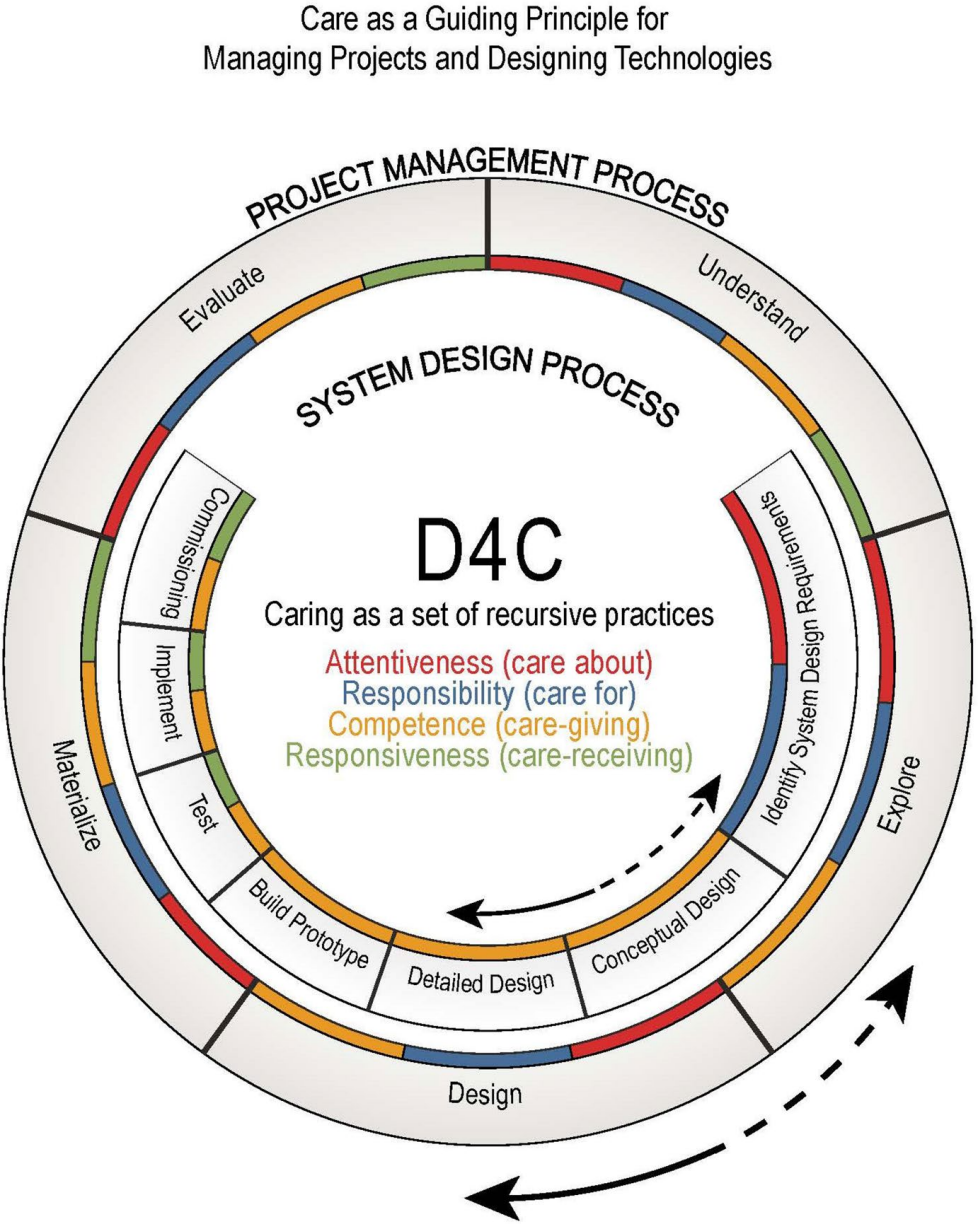
D4C’s four caring practices within both project management and system design processes

Although we build on previous work that argued for the adoption of care in the context of technological design (Damgaard, 2021; Damgaard et al., 2022; Groves, 2009, 2014; Groves et al., 2021; Michelfelder, 2010; Michelfelder et al., 2017; Santoni de Sio & Van Wynsberghe, 2016; van Wynsberghe, 2013, 2016), we propose that D4C may be considered distinctive for several reasons. First, this approach is less interested in embedding values in technological design (as it happens in VSD), but is instead more focused on the exercise of caring practices as dispositions, namely on providing moral guidance to specific actors. Second, D4C can be applied not only in the different phases of the technological design process, but also in the phases of the project management process (see Fig. 1). Third, care as a mid-level guiding principle can help evaluate and prioritize different values at stake within a specific project, thus addressing at least some instances of “value change” (van de Poel, 2018). Interestingly, the value of care in itself may represent an instance of value change according to van de Poel’s taxonomy. Fourth, D4C provides a practice-oriented framework that may influence the management of projects in a way that supports and promotes the fundamental value of care. This approach could be used by the professionals in charge of imagining, developing, implementing, and managing (energy) projects and designing technological artefacts and systems.

The “Approaches to Values in Design” section situates our work in relation to other perspectives about values in design, especially VSD, and clarifies that our proposal builds on existing efforts to use CE in the context of technological design, ethics of technology, and energy research. Section “Values in Energy Systems Design and Innovation” describes how moral values are currently discussed in the context of energy scholarship. Section “Overview and Foundations of CE” presents some of the theoretical foundations of CE while section “Designing for Care (D4C)” illustrates the main assumptions and features of D4C. Finally, in section “A Potential Application of D4C: The Case of a Community Battery Project” we envisage the application of D4C to a community battery project in the town of Rijsenhout, Netherlands, imagining how it could influence the project management as well as the design of this small-scale energy system.

## Approaches to Values in Design

Given the existence of “an intimate relation between technologies and values” (van de Poel, 2015), it is considered possible to consciously and explicitly embed certain “desired” or “right” values into design requirements of technological artefacts and systems (van de Poel, 2013). This idea is well-established in the fields of ethics of technology (van den Hoven et al., 2015), technological design, and innovation (Winkler & Spiekermann, 2018). However, its uptake in the energy transition debate is surprisingly very recent (see the following section “Values in Energy Systems Design and Innovation”).

### Value Sensitive Design (VSD)

To date, the most prominent and used approach in ethics of technology to account for “social”, “moral”, and/or “human” values within the design process is VSD (Friedman et al., 2013, 2017; Winkler & Spiekermann, 2018).^5^ This consists in a “theoretically grounded approach to the design of technology that accounts for human values in a principled and comprehensive manner throughout the design process” (Friedman et al., 2002, p. 1, 2017, p. 63). One of the key contributions of VSD is the detailed development of methodologies in order to design for values. VSD applies a so-called tripartite approach consisting of conceptual, empirical, and technical investigations (Davis & Nathan, 2015). Conceptual investigations aim at identifying stakeholders and giving a first indication of relevant values (Friedman et al., 2013). In empirical investigations, empirical methods are employed to elicit relevant stakeholder values (Friedman et al., 2017). Technical investigations involve the actual design of the technology (Davis & Nathan, 2015; Winkler & Spiekermann, 2018). Although conceptual, empirical, and technical investigations are intended to have the same importance within VSD, and are supposed to be used in an integrative way, empirical and technical investigations seem to play a bigger role in practical VSD studies (Winkler & Spiekermann, 2018). VSD thus seems to take a pragmatic approach to design ethics that is especially appropriate to deal with diverse stakeholders and the values they hold (Albrechtslund, 2007; Manders-Huits, 2011).

However, in relying on empirical methods, VSD has been criticized as lacking “a complimentary or explicit ethical theory for dealing with value trade-offs” (Manders-Huits, 2011), a point that connects also to some types of “value change” presented by van de Poel (2018). Despite being one of the most long-standing and influential approaches to embedding values in the design of technical artefacts, VSD does not have an explicit underlying normative foundation. This is needed in order to guide, for example, the selection of relevant values, distinguish genuine moral values from mere preferences, or provide an indication about how to deal with persistent disagreements among different stakeholders (Manders-Huits, 2011). This criticism has led several authors to suggest that VSD practitioners should explicitly use certain ethical theories or frameworks as tools that can offer additional normative guidance to evaluate values (Albrechtslund, 2007; Jacobs & Huldtgren, 2018). In line with previous work by van Wynsberghe (2013), we suggest that CE can provide such normative foundation and guidance. Differently from van Wynsberghe, however, we posit care as both the foundational value of D4C and also as its guiding mid-level moral principle. Jacobs and Huldtgren (2018) argue that mid-level principle approaches are particularly useful for VSD as they can provide action-guidance in concrete, domain-specific cases and as they converge on the practical level. Other contributions that propose mid-levels principles are found in bioethics (Beauchamp & Childress, 2012) and in the ethics of technology (Peterson, 2017).

### CE in Design Thinking and Innovation

CE has already been applied to VSD in the context of health care (Santoni de Sio & Van Wynsberghe, 2016; van Wynsberghe, 2013) and care for the elderly (Umbrello et al., 2021). In particular, van Wynsberghe (2013) provides an ethical reflection about the role of care robots in promoting the values and the dignity of healthcare patients. She also investigates how Care Centered Value Sensitive Design (CCVSD) can be applied beyond the healthcare domain toward “personal and professional service robots” (2016). While van Wynsberghe adopts Tronto’s moral”qualities” or “elements”, we prefer to use the term “practice”. Other reasons that differentiate our D4C from CCVSD are similar to those discussed above regarding the “distinctive” character of our approach (see the *Introduction*).

More in general, other scholars have used CE in the context of technological design and RRI. For example, Pellé (2016) analyses the normative foundations of responsibility in the recent literature on RRI, including care ethics, in order to illustrate the tension between the modern requirement of pluralism in democratic societies and the applicability of normative theories that demands specific and practical norms to be identified. Michelfelder (2010, 2017) argues that feminist perspectives, including care ethics, allow for envisioning design in different and alternative ways. In particular, Michelfelder et al. (2017) propose “a methodology for a feminist ethics of technology design” that resonates nicely with our proposal as it includes care. Groves (2009, 2014) addresses the fundamental topics of uncertainties related to technological innovation, linking responsibility and care in intergenerational perspectives.

Yet, the most similar attempt to bridge CE and design thinking in the direction we pursue here is represented by the work of Hamington (Flower & Hamington, 2022; Hamington, 2019; Hamington & Sander-Staudt, 2011). In particular, he proposes “Caring Design” as the conceptual, mutually-enriching integration between care and design thinking, stressing that the “relational and responsive dimension of design thinking is analogous in some important ways, namely empathy and inquiry, to the relational and responsive approach of care ethics” (p. 91). Hamington underlines the importance of empathy toward people’s needs and contexts, and advocates for bringing Caring Design into the education of the next generation of business students. Our approach remains in a constructive dialog with these and other authors.

### CE in Energy Research

A few authors have already explored the role and usefulness of care thinking in energy research, especially regarding topics such as vulnerability, energy poverty, access, and justice. Groves et al. (2021), for example, propose that CE’s emphasis “on the moral significance of dependence makes this tradition particularly suited to understanding how detriment can result from the often complex relationships of socio-material dependence that have developed within energy systems” (p. 3). They claim that if relationality provides the “ground of obligation”, and if “social relationships are bound up with power and responsibility”, then CE “can provide firmer foundations for thinking about energy injustice”.

Moreover, Damgaard et al. (2022) underline “relational understandings of energy systems and a language of dependence, necessity and needs as important elements in how people make sense of the energy transition and their place in it.” Hence, they argue that “a language of (inter) dependence, necessity and needs may better reflect people’s own ethical sensibilities” hence “a recognition of relationality and (inter) dependence as basic conditions of existence […]” [p. 1–2, see also Damgaard (2021)]. They suggest that “thinking energy with care” is a way of challenging “the dominance of frameworks and discourses of energy and energy transitions deeply marked by individualism, relying on a language of individual responsibility, rational choice and/or individual rights and justice.”

In the context of energy poverty, Longhurst and Hargreaves (2019) discuss relationships of care as central to understanding the lived experience of the energy poor. Other attempts of energy social science researchers to engage with CE show, for example, that “relational entanglements have a bearing on everyday practices involving energy” (Henwood et al., 2016) and that “people’s social relations influence energy demand” because concrete “energy use occurs in places such as homes, workplaces and communities in which complex webs of social relations already exist” (Hargreaves & Middlemiss, 2020).

## Values in Energy Systems Design and Innovation

Technological design and innovation play a pivotal role within current “socio-technical energy transitions” (Büscher et al., 2019). As the expression “socio-technical” suggests, these complex transformations include human and social dimensions. These play a role in how (energy) technologies and systems are designed. For example, they influence (1) how they are conceived and realized; (2) how they are used; (3) how they affect the energy cycle (i.e., generation, distribution, use, waste); (4) how they affect the wellbeing of users and the integrity of broader socio-ecological systems.

Certain values (e.g., justice, accessibility, affordability, sustainability, cleanness) may be relevant for the design of energy technologies and systems. For example, Levenda (2019) propose “energy values” as an analytical concept to elucidate differences in the context of energy innovations. Milchram et al. (2019) highlight the role of values in the analysis of energy systems, claiming for example that electricity systems such as smart grids can be design *for* justice (Milchram et al., 2020). Moreover, few authors have considered how values play a role in specific energy projects such as offshore wind farms (Künneke et al., 2015), smart grids (Milchram et al., 2018), and shale gas explorations (Dignum et al., 2016). To anticipate our example, the design of a community battery may promote values among the system’s users such as solidarity and cooperation or, on the contrary, individualism and competition.

Any concrete energy project involves individuals and groups that, besides having different and sometimes overlapping roles, all hold certain moral values and are typically capable of making moral judgements.^6^ There are at least three main groups of stakeholders and actors within most (energy) projects: (1) *Citizens and users*: people and non-human beings affected by the project; (2) *Practitioners*: designers, engineers, managers, or the professionals in charge of envisioning, developing, implementing, and managing the project; (3) *Institutional and governmental actors*: people and institutions that represent and provide the political and legal frameworks within which the project occurs (e.g., municipalities, governmental bodies, utility companies).

In the context of energy projects, practitioners and institutional actors stand in a peculiar position. They have a specific status, a defined role, and often a moral reference point in a professional ethics (e.g., code of conduct, moral protocol). Their authority and influence over the course of the entire project development and design process imply clear hirearchies which may involve asymmetries in power dynamics and hence additional responsibilities. Beside personal values, they must adhere to certain *professional* values such as safety, trust, and integrity, depending on their professional expertise and on the existence of ethical guidelines, protocols or codes of conduct. These professionals should be (at least) keenly aware of the existence of citizens’ and users’ values and strive to know about them by involving social science researchers from the initial stages of any project that affect citizens and/or users. These actors should be capable of gauging, balancing out, and evaluating citizens’ and users’ values, professional values as well as their own personal values. In particular, practitioners face a moral conundrum: How can they effectively evaluate and responsibly decide which values should be prioritized and taken into account in the case of a value conflict or trade-off? How can they identify and address instances of “value change” (van de Poel, 2018)? Of course, they could set some arbitrary rules or criteria, follow their own moral intuitions, or adopt ethical protocols. As we will discuss below (Section “Imagined Potential Changes due to Adoption of D4C”), our proposal is that D4C offers an alternative practice-oriented approach to deal with values and address some instances of “value change” within projects. The following section introduces some historical developments of care thinking and a few theoretical foundations of CE.

## Overview and Foundations of CE

According to a traditional account, CE emerged in rather antagonistic terms as a feminist response to scholars defending more established ethical theories (e.g., deontology or utilitarianism). Care ethicists criticized these theories for being too “idealized” given their ambition to provide general norms and standards of conduct (Norlock, 2019). In contrast, CE would be “rooted in receptivity, relatedness, and responsiveness” (Noddings, 1984, p. 2) that emerge from concrete experiences and relationships. In this sense, CE “emphasizes dimensions of morality evolved from feminist^7^ theory […]” (Hamington & Sander-Staudt, 2011, p. ix). Moreover, Held and Tronto underline the connection between care and social justice (Held, 1993, 1995, 2006; Tronto, 1993, 2013), stressing the decisive political significance of care (see also, e.g., The Care Collective, 2020). In any case, today care ethicists seem less interested in defending boundaries and creating contrapositions. Therefore, although it was “bolstered by sometimes critical feminist writings on care throughout the 1980s and 1990s, feminist scholars and non-feminist scholars are increasingly taking care ethics seriously, applying it to social and political issues, including questions about business ethics […]” (Hamington & Sander-Staudt, 2011, p. ix).

Care is “difficult to define because of its vast connotations” (Hamington & Sander-Staudt, 2011, p. viii) and the use of different terms and notions.^8^ However, authors tend to agree that “care theory is based on the notion that humans are fundamentally relational, existing in a dynamic web of associations” (Hamington, 2019, p. 92). As anticipated in the introduction, we will follow here Hamington’s definition of care. Moreover, we adhere to an ambitious notion of political *universal care*, “the ideal of a society in which care is placed front and centre in every scale of life. Universal care means that care—in all its various manifestations—is our priority not only in the domestic sphere but in all sphere: from our kinship groups and communities to our states and planet” (The Care Collective, 2020, p. 19).

Within D4C, “care” (noun) represents both a foundational value and a mid-level guiding principle. In addition, “caring” (verb) indicates the concrete exercise of concrete (recursive) caring practices. Of course, CE has received several criticisms too (Norlock, 2019). For example, it might lack a comprehensive and systematic theoretical account. It might overstress the burdened history of femininity, which in turn may damage a feminist agency (e.g., exploitation of caregivers). Moreover, identifying CE exclusively within feminine thinking may distract from women’s capacity to harm, since not all women prioritize care.

Four assumptions present in CE thinking are especially relevant for D4C:

1. Relational ontology.
2. Vulnerabilities and dependencies.
3. Agency.
4. Ecological turn.

(1) *Relational ontology of individual and group identities*. Several CE thinkers criticize the idea of an abstract subject defined as completely autonomous, rational, and neutral as the “fairly entrenched belief in Western individualism” (Baier, 1987, p. 48). According to this view, the western tradition of thought has been primarily considering “individual human beings as social atoms, abstracted from their social contexts, [disregarding] the role of social relationships and human community in constituting the very identity and nature of individual human beings” (Friedman, 1989, p. 275). By contrast, a “relational” conception of identity acknowledges “the role of social relationships and human community in constituting both self-identity and the nature and meaning of the particulars of individual lives” (Friedman, 1989, p. 276).^9^ When self-identities are inescapably and constitutively understood as bundled within networks of relationships, the notion of “self” becomes a “network self” (Wallace, 2019). This notion lends itself to fascinating analogies of webs or networks of relationships, which are well-suited to illustrate the bundle of relations and interconnections typical of social media, the internet, but also energy systems (e.g., electric grids). As Damgaard et al. assert, “notions of care within energy research ought to be associated with a recognition not only of the social relations within which energy practices are embedded, but also of their significance as emotional engagements, challenging the primacy of rationalist framings of behaviour, choice and forms of engagement” (2022, p. 3). Although relational ontologies are not exclusive to CE, “care is governed by and enacted through a relational ontology” (Doucet, 2017, p. 15).

(2) *Recognition of vulnerabilities and* (*mutual*) *dependencies*. CE recognizes the situatedness of personal circumstances as well as individuals’ degrees of vulnerability.^10^ Morally speaking, this implies the duty to understand different lived experiences and contexts in order to be able to protect and promote the interests of those involved. Such sensitivity for the context dependent features of irreducibly particular situations (i.e., economic, socio-political, and environmental circumstances) renders persons specific histories and identities central and non-universalizable (Jaggar, 1995, p. 186). CE does not only take concrete human relationships as key starting points but also the asymmetries of (many of) these relationships (e.g., dependencies and patterns of interconnection). Indeed, moral relations occur not only between equals (who have voluntarily entered that relationship), but also among those *not equally situated or empowered*, individuals who find themselves in relationships that they themselves may *not have chosen* (as children find themselves in relation to parents) (Tronto, 1993, 2013).^11^ The existence of asymmetries among unequals is relevant and applies in the case of energy projects too. For example, cobalt mining is crucial for batteries production but it has been shown to represent a paradigm of unequals [i.e., multinational companies vs. vulnerable miners, see e.g., (Sovacool, 2020)].

(3) *Agency*. Notions of agency and responsibility are underpinned by that of identity or selfhood. As Gilligan puts it, as “a framework for moral decision, care is grounded in the assumption that self and other are interdependent, an assumption reflected in a view of action as responsive and, therefore, as arising in relationship rather than the view of action as emanating from within the self” (Gilligan, 1993, p. 471). For Pettersen, this view allows for “a wider understanding of who the moral agents are […where] relationships transcend boundaries separating the private from the public, the individual from the collective” (Pettersen, 2011, p. 53). Similarly, Robinson stresses that “the relational ontology of a critical feminist ethics of care—which emphasizes human interdependence and mutual vulnerability—overcomes the dichotomies between the needy and the strong, victims and agents, and objects and subjects […]” (Robinson, 2011, p. 18). In this perspective, moral deliberations require not only reason, but also empathy, emotional responsiveness, and perceptual attentiveness. Although blurring the lines between identity and agency may cause confusion in attributing individual responsibilities (e.g., in the context of law infringement), Kittay invites to understand moral harm “*less* [as] *a matter of the violation of rights*, and more [as] the consequence of *failures in responsibility and responsiveness* […]” (2011, p. 53).

(4) *Ecological turn.* Concrete (energy) projects take place within ecological systems. Drawing from a recent proposal to embrace an “ecological turn” in CE (de la Bellacasa, 2017; Pierron, 2019), we agree that caring practices should be understood within projects’ ecological situatedness, extending caring from intra-species to inter-species relations. De la Bellacasa affirms that the “generation” of care in “strongly stratified technoscientific worlds” is both shortsighted and difficult. She proposes that “generating care means counting in participants and issues who have not managed or are not likely to succeed in articulating their concerns” (p. 94). According to this view, “matters of care” do not only refer to human participants, but also to non-human entities and beings that she defines “neglected things”.^12^ In this sense, an ecological CE attempts “the subversion of anthropocentrism and anthropomorphism, in favor of de-centered and distributed agencies in these ecological matters of care” (Brons, 2019). Given that energy projects typically take place in an ecological space and have always some kind and degree of environmental impact, conceiving care in ecological and non-anthropocentric terms is theoretically pertinent and morally forward-looking. Consider, for example, the material and energy intensity within the life cycle of a technology (i.e., from resources extraction to recycling and disposal). Adopting an ecological perspective means always considering an energy project within an ecological system (e.g., ecoregion), which implies concrete consequences. The environmental impact assessment would become more demanding, so that interspecies and ecological relations must be taken into account from the initial steps of any project. Any relevant harm inflicted on the non-human beings living in the ecological system of reference would require concrete mitigation and compensation measures. For example, rewilding projects (Pereira & Navarro, 2015; Perino et al., 2019) may represent substantial ways of integrating care in ecological restoration and conservation^13^ (e.g., the construction of a wind farm requires that of wildlife crossings, or the creation/extension of a protected areas). These four assumptions of CE may not be shared by all care ethicists. Yet, we consider them foundational for the type of CE that informs and supports D4C.

## Designing for Care (D4C)

### Care as a Foundational Value and a Guiding Mid-level Principle

Drawing from the previous section, D4C is based on the following assumptions and premises:

1. *Care* as foundational *value* and mid-level guiding *principle*.
2. Distinction between *care* as both *value* and *principle*, and *caring* as a *process*.
3. Caring process made of a set of recursive *caring phases* and *practices*.
4. Relational ontology of individual and group identities.
5. Ecological perspective.

Drawing from the previous discussion (Section “Overview and Foundations of CE”), we propose that D4C is centered on the foundational value of care, which is also upheld as a mid-level guiding principle.^14^ Care is enacted through recursive caring practices which are part of a caring process. Care and caring are based on a relational ontology and an ecological perspective. They depend on spatio-temporal contexts as well as on the specific people involved. Care is therefore envisioned as a value that meets the need of a political good as “collective caring” (Engster, 2005).

As a *mid-level* principle, care is *relative* (rather than a *high*, *general*, or *absolute* principle) because it belongs neither to high theory nor to mere practice but speaks to both. As a principle, care can provide moral orientation and guidance within both project management and technological design processes, influencing various relationships (i.e., between designers, managers and users, and among users). Moreover, we suggest it can help assess, evaluate and judge the different personal and group values held by citizens, users, designers, institutional and governmental actors that take part in a specific project. This could become especially useful when instances of “value change” emerge. In this case, care as a mid-level guiding principle functions as a “filter”, allowing, prioritizing, and integrating in both processes (again management and design) only the values that are compatible with the foundational value of care and with the exercise of the (four) caring practices.

### The Caring Process and Its Practices

Fisher and Tronto introduce the idea to articulate caring as phases (1990). Tronto further expanded on this by describing the four “moral qualities that align with the four phases of care” (1993). Drawing from these authors, we propose that D4C caring process becomes visible through central *caring practices*. Besides the main four practices discussed below, later we will mention additional ones that are compatible, and could therefore be integrated, within D4C. These caring practices extend over time, they belong to caring phases (which could also be defined as “steps” or “dimensions” of the caring process).^15^ These phases and their corresponding practice should be conceived as sometimes overlapping, interwoven, and often recursive. It goes without saying that both the project management process as well as the system design process include different phases that could also be recursive^16^ (consider the arrows in Fig. 1). On the one hand, there is the overall *energy project management process*, which involves all three groups of actors described above. This process begins with the existence of a need (e.g., providing electricity access) or problem to be solved (e.g., realize a renewable off-grid energy system). The (energy) project management process includes different phases: understand, explore, design, materialize, and evaluate (see, e.g., Pahl et al., 2007).^17^ This process depends on project manager(s), who are obviously the actors primarily concerned with the exercise of the four caring practices in the project management process. On the other hand, there is the *system design process*, which involves primarily designers and engineers and is characterized by several and sometimes recursive phases: identification of system design requirements, conceptual design, detailed design, building of a prototype, testing, implementation, and commissioning. Although all stakeholders are affected by the project, only some actors can play a central role within each process. The caring practices mentioned in the center of the graph (Fig. 1) should influence different phases within both processes, hence the practical behavior of the actors who are in charge of each phase/process.

Let us now consider D4C’s four main caring practices in more detail:

(1) Practice: *Attentiveness*—Phase: *Caring about.*

Tronto states that “at this first phase of care, someone or some group notices unmet caring needs” or the existence of a problem which calls for attentiveness, intended as the temporary “suspension of one’s self-interest, and a capacity genuinely to look from the perspective of the one in need” (Tronto, 2013, p. 34). Although attentiveness is primarily directed toward others, it also refers to one’s own needs (Cf. below, *self-care*). In an energy project, practitioners should not only be interested in listening to other stakeholders, but should also encourage an actual participatory process (Velden et al., 2014). Secondly, it means that practitioners should develop strategies to work with stakeholders and capture their priorities and preferences, usually by involving social scientists during the initial stages of the project. For managers, attentiveness is relevant throughout the process. Designers’ attentiveness is especially important in the first phase of the design process.

(2) Practice: *Responsibility*—Phase: *Caring for.*

Tronto affirms that “once needs [or problems] are identified, someone or some group has to take on the burden of meeting those needs. This is responsibility, and that is the key moral quality of this second phase” (Tronto, 2013, p. 34). Here, responsibility is especially relevant for the professionals who manage project developments, design energy systems, govern public interests and create policies. Other authors support the interpretation of responsibility as care (Pellé, 2016), especially relevant in an intergenerational perspective (Groves, 2014). Practitioners within any energy projects should The exercise of responsibility is central to all practitioners conduct. In energy projects, managers should act responsibly in all the phases of the project management process. Designers should especially be responsible within the first phase of the system design process.

(3) Practice: *Competence*—Phase: *Care-giving.*

In practice, care givers should be morally competent in delivering care. Tronto writes: “Assuming responsibility is not yet the same as doing the actual work of care; doing such work is the third phase of caring and requires the moral quality of competence. To be competent to care, given one’s caring responsibilities, is not simply a technical issue, but a moral one” (Tronto, 2013, p. 35). Although competence is primarily rooted in moral experience and capacity, in our case actual care-giving can be understood as a moral competence that informs professional expertise (e.g., design, engineering) and related practices. Providing concrete care means that managers will try to exercise it through all phases of the management process. Designers should exercise care-giving in all phases after the identification of system design requirement, which means integrating care within their professional skills.

(4) Practice: *Responsiveness*—Phase: *Care-receiving.*

Anticipating an ecological expansion of CE, Tronto affirms: “Once care work is done, there will be a response from the person, group, animal, plant, environment, or thing that has been cared for. [This means] Observing that response, and making judgments about it (for example, whether the care given was sufficient, successful, or complete” (Tronto, 2013, p. 35). Although “some response is necessary” (p. 35) for the caring process to work and continue, some care-receivers may not be able to respond in a direct and intelligible way (e.g., plants, ecological systems). In such cases, however, ecological sciences can determine the concrete impacts of care-giving and thus provide an informative response. In an energy project, responsiveness will primarily come to practitioners as feedback/reactions from the users.

As anticipated above, there are other important caring practices that might play a significant role within D4C. For example, the practices of (5) *solidarity* (phase: *Caring-with)* and (6) *self-care* appear as preconditions for the other practices to be carried out. On the one hand, Tronto states that solidarity is underpinned by and depends on specific values: *plurality*, *communication*, *trust*, and *respect*. This implies that “caring needs and the ways in which they are met need to be consistent with democratic commitments to justice, equality, and freedom for all”. Tronto’s suggestion is that “in order to think about democratic care”, caring-with “requires a change in the values of citizens” so that they “care enough about caring—both in their own lives and in the lives of their fellow citizens—to accept that they bear the political burden of caring for the future” (Tronto, 2013, p. xii). The other prerequisite of a caring process is self-care. Kittay (2011) underscores it as a crucial phase that focuses on the needs and problems of care-givers. For Kittay, self-care is particularly relevant within professional settings (e.g., hospitals) or in the context of family life [e.g., postpartum (but not only) mothers]. Other caring practices that we will not discuss in this paper but could inform D4C are: (7) *benevolence*, (8) *empathy* and *sympathy* (or *compassion*), (9) *cooperation*, and (10) *reciprocity*. In the last section, we imagine how the adoption of D4C may influence the project management as well as the design of a small-scale energy system.

## A Potential Application of D4C: The Case of a Community Battery Project

### Description of the Project and Its Implementation

**Fig. 2:**
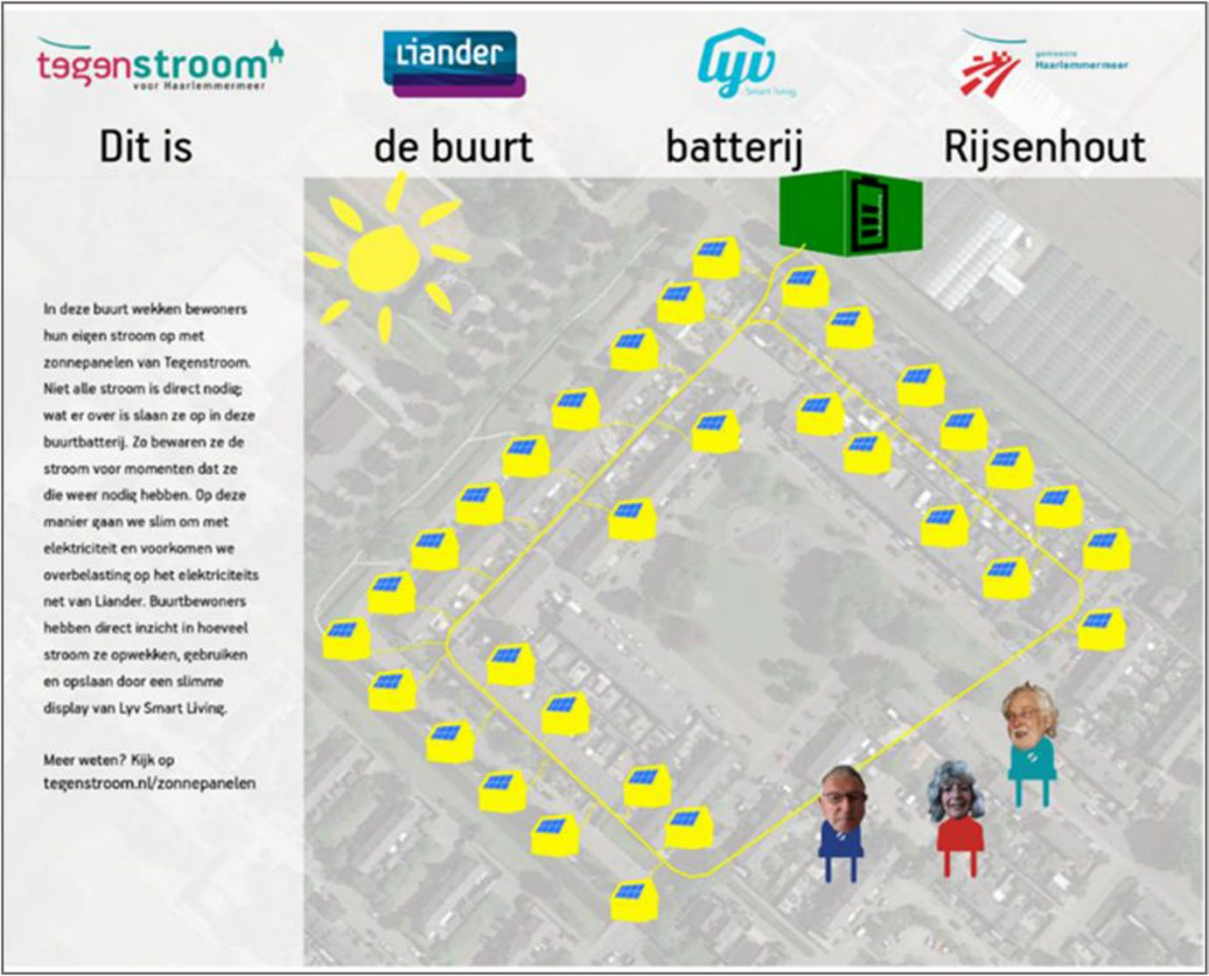
Excerpt of a brochure that depicts the project. Source: Tegenstroom

In order to envisage how the adoption of D4C might affect the project management and the technological design processes, we apply it a posteriori D4C to the case of an existing community battery project in the Netherlands. Community battery systems (CBS) are electricity storage systems located in a spatially defined neighborhood and connected to multiple households. The intention is to store renewable electricity, which is typically generated within a neighborhood, e.g., through solar photovoltaic (PV) systems (Kalkbrenner, 2019). Such batteries are thus used to bridge renewable energy intermittency, or the temporal gaps between electricity generation and use in a neighborhood. This was also the rationale for the specific CBS that we use here to illustrate the concrete effects of adopting D4C [cf. (2020) for a detailed analysis of this case]. Implemented in the suburban village of Rijsenhout near Amsterdam, the CBS was a pilot project jointly promoted by the local distribution system operator (DSO), the municipal energy supplier and a provider of energy management software (see Fig. 2). DSO initiated the project with the aim of testing how a community battery could be used to store solar supply peaks and stabilize the voltage in the local low-voltage network.

**Fig. 3:**
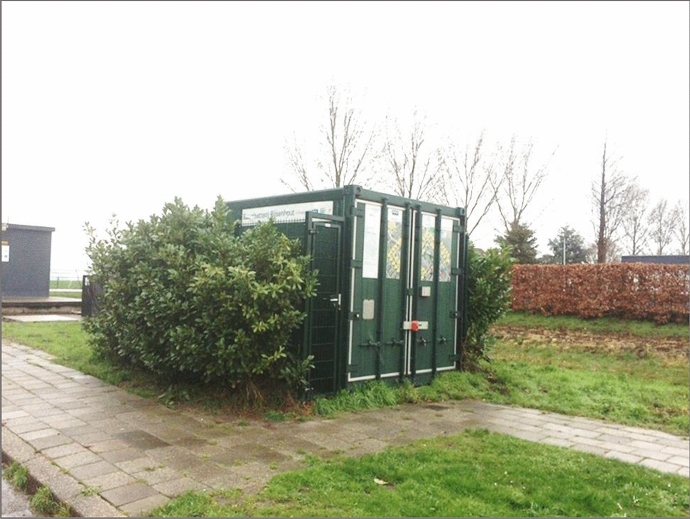
The external appearance of the community battery. Source: Author

The neighborhood was chosen partly because of technical characteristics (a large number of PV systems in a small space) and partly because of the pre-existing business relationship between the municipal energy supplier and the households. The pilot system consisted of 35 households (tenants of a social housing corporation) with PV systems (rented from the municipal energy supplier for 30 euros/month), the CBS, smart metering, a software to (dis)charge the battery, and an app for the households to show electricity generation, use, and storage. The CBS was placed in a cargo container at a street corner in the neighborhood (see Fig. 3). Each of the 35 households had access to a fixed battery capacity of 3 kWh. The battery (dis)charging process was optimized such that the largest daily supply peaks were reduced and self-consumption for the households maximized. Input for the optimization were demand and supply forecasts, i.e. forecasting households’ use of electricity from smart meter data and forecasting solar generation dependent on weather forecasts. Households could not “access” the battery via the app and change battery settings. In exchange for participating in the project, households received a 50% discount on their PV system rent for 1 year. They did not have to pay for the installation of the battery and the smart meters as these costs were covered by the project partners and a subsidy from a national agency.

Overall, both the project development and the design process had a top-down approach. The DSO owned and operated the battery. The local energy supplier was responsible for communicating with the households and the billing system. The system was mainly designed by the DSO, and it was only after its completion that the energy supplier recruited households for participation. Households could only influence decisions on the aesthetics of the battery container: the color, the exact location, and the planting of bushes around it.

### Imagined Potential Changes due to Adoption of D4C

Viewing this energy development through the lens of D4C would potentially transform both how the project was envisioned and how it was carried out. Relational ontology would point out that this specific socio-technical system is not only the community battery and its grid but it includes also the people who design, build, and eventually use it. All of them are part of a network of relationships that, in the case at hand, could have led to different kinds of relationships, communication, and engagements. Considering the project from the standpoint of ecological caring implies that the design and construction of the community battery would have required not only a rigorous environmental impact assessment, but also measures for mitigating and compensating potentially affected non-human beings and the local ecosystem. Table 1 illustrates how D4C could have affected the various relationships existing as well as the project management (or governance) and the technological design, in all likelihood making these processes more inclusive and bottom-up. The first two columns correspond to the different caring practices. Then, we describe possible transformations for the designers, the users, the system design, and the project management. The final column reports the project and design phases that are affected according to Fig. 1. Within this specific project, we suggest that D4C may offer moral guidance to overcome difficulties arising in at least three types of value change described by van de Poel (2018) in his taxonomy (especially, types 2, 3, 4, see above).

**Table 1:**
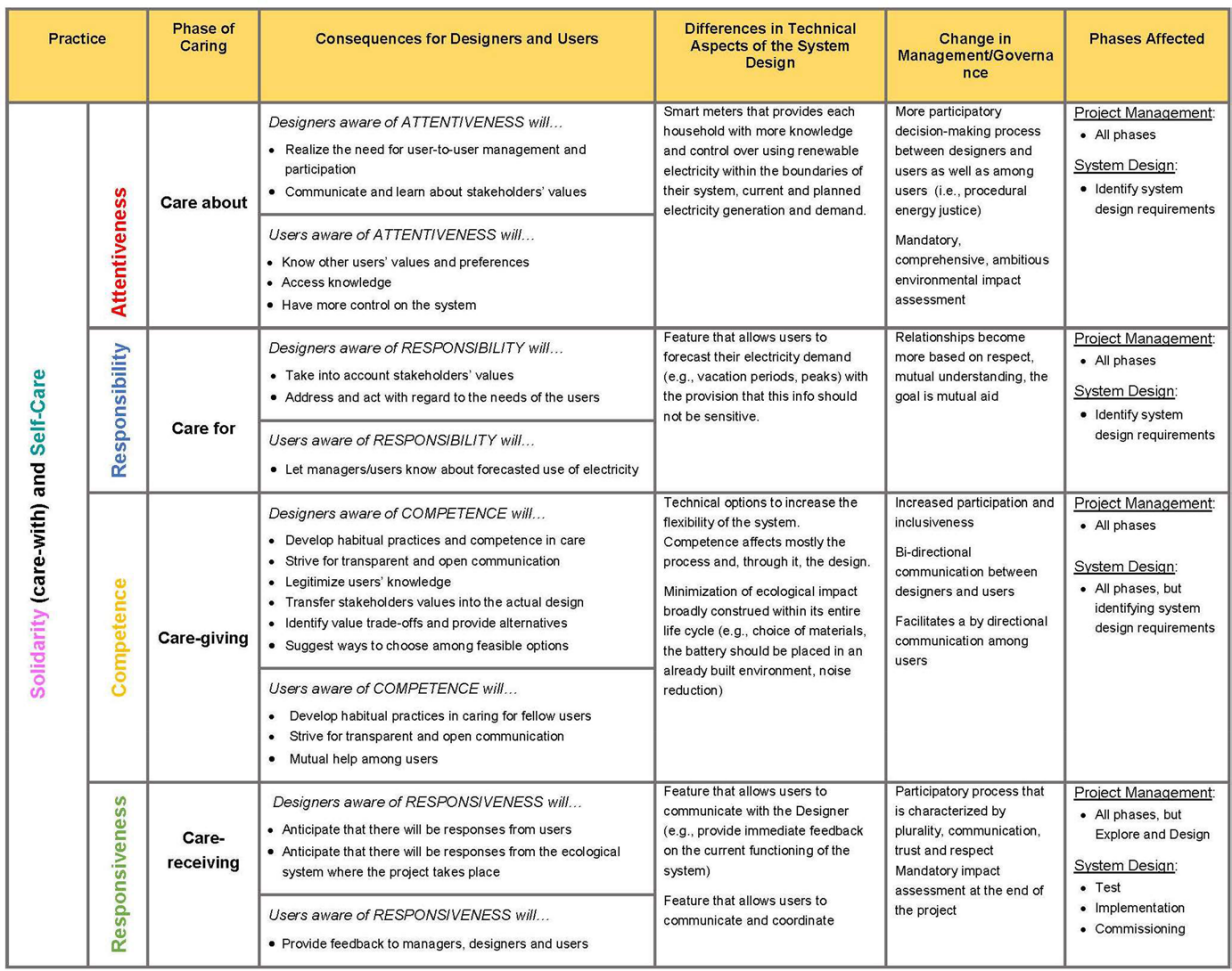
Potential Effects of Adopting D4C in the Case of a Community Battery

## Conclusion

*Designing for Care* (D4C) is proposed as a distinctive approach to project management and technological design informed by Care Ethics. Rather than arguing for embedding certain values (e.g., care) in the design of technological artefacts, we argue for a practice-oriented approach that aims to influence the mentality and the practices of the professionals in charge of managing (energy) projects and designing technological artefacts and systems. On the one hand, D4C suggests centering management and design on the fundamental value of care, which in D4C becomes also a mid-level guiding principle. On the other hand, practitioners who adopt D4C would agree with some of its assumptions. For example, they would stress the importance of understanding (human) life and its relationships according to a relational ontology. They would envision “a different normative approach [that] could pave the way for a more caring environment” (Pettersen, 2011, p. 53), because in D4C the design and production of technological artefacts and systems are conceived within ecological systems and respectful of their limitations and well-being. Practitioners would concretely try to enact a set of caring practices within both processes as part of their moral dispositions. This would inevitably challenge longstanding assumptions present in more traditional approaches to management and design. At the same time, they would embrace D4C as a prescriptive approach, thus addressing and contributing to overcome the normativity gap about values in design. Although this paper discusses only one energy project, D4C may be adopted and tried in other contexts, sectors, and fields. This theoretical contribution is an initial attempt to articulate the structure and the procedural character of D4C. The application of D4C in a concrete project is needed to assess its actual impact, benefits, and limitations. The article aims to contribute to the debate about CE in engineering practices and especially to improve the work of all those practitioners (i.e., project managers, designers, engineers) who have the responsibility to conceive, design, and implement energy projects in the context of current energy transitions.

## Footnotes

1 In the title, we privilege “designing” rather than “design” to stress the importance of caring as process in time made of recursive phases. We do not claim to present a fully-fledged and detailed account of “Designing for Care”. This is an initial attempt to articulate the structure and the procedural character of D4C.

2 Similarly to the “caring process”, both the “management of (energy) projects” and “technological design” are assumed to be processes all of which are mad of phases. More specifically, the former deals with the steps of actually envisioning, designing and implementing (energy) projects. The latter refers primarily to designing sociotechnical (energy) artefacts and systems.

3 Here, care is not understood as a “first” or “high” principle but rather as a “mid-level” principle among others that might be relevant in the context of managing and designing. In other words, the principle of care provides the initial criterion for determining whether actions, choices and decisions (which depend on certain values) are helpful to the self and to others, are attentive of their needs and interests, responsible, competent and responsive in providing and receiving caring deeds.

4 They write: “On the most general level, we suggest that caring be viewed as a species activity that includes everything that we do to maintain, continue, and repair our ‘world’ so that we can live in it as well as possible. That world includes our bodies, our selves, and our environment, all of which we seek to interweave in a complex, life-sustaining web” (Fisher & Tronto, 1990). Both definitions underscore the general CE proposition that care “involves maintaining the world of, and meeting the needs of, ourself and others” (Sander-Staudt, 2020).

5 Other approaches, which broadly share the same aim, are “Design for Values” (Velden et al., 2014; Van den Hoven et al., 2015; Dignum et al., 2016), “Values in Design” (Flanagan et al., 2008), as well as some value-specific versions such as “Design for Privacy”, “Safe-by-Design” or “Design for Wellbeing” (Jenkins et al., 2020).

6 This may be the case in general, but some stakeholders may not be able to do so. As we will see, D4C will be particularly valuable in situation where participation is compromised for people with disabilities and/or people who are perceived to be disabled, that is when participation is difficult or limited.

7 However, CE should neither be considered as only and necessarily tied to feminist accounts nor imply that care is primarily a feminine notion.

8 For example, Noddings suggests that it is a “set of dispositional attitudes” (1984), Tronto privileges the term “practice” (1993), Ruddick defines it as “a labor that is inherently relational” (Ruddick, 1995). Halwani (2003) argues to subsume CE under virtue ethics by constructing care as a “virtue”. Slote employs the expression “a motivational virtue” (Slote, 2007). Indeed, the fact that care requires habitual practice and repetition make it compatible with virtue ethics (Halwani, 2003) and also with the Capabilities Approach (Groves et al., 2021). Miller and Bernasconi suggest that it is “a different way of thinking about duties” (2011), and Hamington (2004) claims that it is “a completely novel approach to morality”. Moreover, “many care ethicists define care most fundamentally as a face-to-face practice to underscore that care is work that must be done on a direct level, and that involves energy expenditure and personal investment on the part of its practitioners” (Hamington & Sander-Staudt, 2011, p. viii).

9 For Robinson, “the relational ontology of care ethics claims that relations of interdependence and dependence are a fundamental feature of our existence” (2011, p. 4). For Tronto, “the world consists not of individuals who are the starting point for intellectual reflection, but of humans who are always in relations with others. To make sense of human life requires a relational perspective […] so that claims made about individuals that do not place them in a relational setting will be incomplete” (2013, pp. 36–37).

10 This often means that the degree of moral consideration should vary and proportionality should reflect the degree of vulnerability of those affected. Although this trait of CE has been criticized for being an instance of partisanship that violates impartiality in ethical judgements (i.e., care ethics can promote favoritism which violates fairness, e.g., moral responsibility toward strangers), it still coheres with the idea that care in practice requires the establishment of specific and often lasting caring relationships.

11 In this sense, ethics is not, at least not primarily, concerned with a freely chosen interconnection between equals but, for example, “a connection between a child and her unchosen mother and father, or a child and her unchosen older and younger siblings, or indeed between most workers and their unchosen fellow workers, or most citizens and their unchosen fellow citizens” (Baier, 1987, p. 48).

12 Besides being thought-provoking as a theoretical proposal, this “ecological turn” in CE resonates with other attempts pursued in the field of (eco)feminist environmental ethics. For example, authors such as Plumwood (1993), Warren (1997) and Haraway (2008) spelled out the complex relationships between human persons and non-human lives (e.g., plants and animals) and existences (e.g., ecological assemblages such as rivers, watersheds, ecoregions, ecosystems).

13 In this regard, see also the Feral Atlas project (Tsing et al., 2021).

14 Care as mid-level principle is similar to the principles proposed by Beauchamp and Childress (2012) or Peterson (2017). Peterson clarifies that mid-level principles “are less general than high-level ethical theories, but not as specific as moral judgments about particular cases” (p. 8).

15 It is important to note that although we use “practice(s)”, other scholars have used different terms, for example, “virtues”, “elements” or “qualities”. For instance, van Wynsberghe writes that “These phases have corresponding moral elements as standards to evaluate the care practice from a moral standpoint” (2013, p. 418).

16 What we previously called “technological design process” is described in Fig. 1 as “system design process” because here we are applying D4C to a specific energy system rather than a specific technology (i.e., device).

17 See also: https://hpi.de/en/school-of-design-thinking/design-thinking/what-is-design-thinking.html
